# The identification of pregnant women with renal colic who may need surgical intervention

**DOI:** 10.1186/s12894-022-00985-x

**Published:** 2022-03-07

**Authors:** Maomao He, Xiaoting Lin, Ming Lei, Xiaolan Xu, Zhihui He

**Affiliations:** 1 Department of Obstetrics and Gynecology, The First Affiliated Hospital of Guangzhou Medical University, Guangzhou, 510120 China; 2 Department of Urology, Minimally Invasive Surgery Center, The First Affiliated Hospital of Guangzhou Medical University, Guangdong Key Laboratory of Urology, Guangzhou, 510230 China

**Keywords:** Renal colic, Pregnancy, urolithiasis, Intervention

## Abstract

**Background:**

Renal colic is a surgical emergency in pregnancy that is caused by a range of non-obstetric factors and known to occur more frequently during the second and third trimesters. Several studies have reported that up to 70–80% of stones pass spontaneously during pregnancy. There are some patients will not pass their stones and will ultimately require surgical intervention. Through retrospective analysis of the clinical data of 212 pregnant women with renal colic, the predictive factors of pregnant women with renal colic in need of surgical intervention were determined.

**Methods:**

We conducted a retrospective review of 212 pregnant women presenting with renal colic between 1st January 2009 and 31st December 2020. Univariate and multivariate analyses identified a range of predictive variables for surgical intervention. In addition, we used receiver operating characteristic curve analysis to evaluate the predictive power of our model and created a nomogram for clinical application.

**Results:**

Of the 212 patients presenting with acute renal colic in pregnancy, 100 patients (47.2%) underwent surgical intervention and 112 patients (52.8%) were treated conservatively. Univariate analysis identified significant differences between the two groups with regards to fever, the duration of pain, white blood cells, C-reactive protein, ureteral stone size, hydronephrosis, and stone location. Multivariate analysis further identified a number of independent predictors for surgical intervention, including fever, a duration of pain ≥ 4 days, a ureteral stone size ≥ 8 mm, and moderate or severe hydronephrosis.

**Conclusions:**

We identified several independent predictors for surgical intervention for renal colic in pregnancy. Fever, a duration of pain ≥ 4 days, a ureteral stone size ≥ 8 mm, and moderate/severe hydronephrosis, play significant roles in predicting surgical intervention. Our nomogram can help to calculate the probability of surgical intervention in a simple and efficient manner. Prospective studies are now required to validate our model.

## Background

Renal colic is a surgical emergency in pregnancy that is caused by a range of non-obstetric factors and known to occur more frequently during the second and third trimesters. Approximately 1:200 to 1:1500 pregnant women experience a symptomatic stone event that might be caused by renal colic or complications related to renal colic [1, 2]. There is no significant difference between pregnant and non-pregnant women with regards to the probability of developing renal colic [3]; however, this condition is potentially troublesome and can lead to hospitalization, invasive treatment, and serious adverse effects for both the mother and fetus.

Several studies have reported that up to 70–80% of stones pass spontaneously during pregnancy. This is partly because the urinary tract is normally dilated in pregnant women, although a previous study reported that 50% of such patients expel their stones during the post-partum period [4]. However, the rate of spontaneous stone passage ranges from 48 to 84% in the existing literature and should not really differ from the rate of spontaneous stone passage in non-pregnant women when stratified by stone size [5–7]. There are some patients will not pass their stones and will ultimately require surgical intervention. These patients may suffer from unrelenting colic pain, premature rupture of membranes [8]. and preterm delivery [9], renal impairment, and even septicemia.

Therefore, there is an urgent need to develop a method that could identify patients who are more likely to require surgical intervention. This would enable clinicians to optimize their management plans and minimize the impact of renal colic on both the mother and fetus, thereby reducing readmission, hospitalization time, and costs. Previous studies have identified a number of factors that show promise for predicting the probability and duration of spontaneous passage, including the location and size of the calculus, pain, the degree of hydronephrosis, perinephric stranding, white cell counts [10–12]. Very few studies have specifically considered the prediction of surgical intervention for renal colic during pregnancy. The aim of this study was to identify predictive factors that could pregnant women who are likely to require surgical intervention during the acute presentation of renal colic.

## Methods

### Study subjects

This was a retrospective review involving pregnant patients with renal colic at The First Affiliated Hospital of Guangzhou Medical University between 2009 and 2020. All procedures performed in studies involving human participants were in accordance with the ethical standards of the institutional at the First Affiliated Hospital of Guangzhou Medical University (The version number was V 1.0) and with the 1964 Helsinki declaration and its later amendments or comparable ethical standards. Informed consent was obtained from all individual participants included in the study. The study population included pregnant women who were treated for renal colic in hospital. The diagnosis of renal colic and underlying urolithiasis was based on the clinical manifestations of pain, location, the time of onset, and ultrasound findings. We excluded cases involving abdominal pain that was caused by other factors. When the process of clinical management was unclear on the medical records, or if clinical data were absent, we contacted the patients involved by telephone and retrieved the missing information. We excluded patients if the method of intervention was unclear. The cohort was divided into patients who underwent surgical intervention (ureteral stent placement, nephrostomy or ureteroscopic lithotripsy; Group 1) and those who were managed effectively by conservative methods, as demonstrated by the lack of symptoms during pregnancy on follow-up and normal levels of creatinine in the blood (Group 2).

### Data collection and variables

We recorded a range of data for each patient, including patient demographics (fever, including body mass index [BMI], the duration of pain, indicators of infection (white blood cells [WBCs], C-reactive protein [CRP]), stone-related parameters (size and location) and hydronephrosis. Fever referred to Body temperature over 37.5 °C. The duration of pain referred to time from the beginning of pain to attendance at the emergency room. Stone size was based on the largest meridian of the stone after ultrasound measurement.

Stone location was divided into three categories: kidney, ureter, and none. Data relating to hydronephrosis was acquired by ultrasonography of the urinary system. Hydronephrosis was classified as mild(< 30 mm), moderate(≥ 30 mm, < 40 mm), and severe ≥ 40 mm).

The first-line management of patients who developed renal colic was conservative, including hydration, painkillers, and antibiotics. Surgical intervention was required if conservative therapy failed or the patient suffered any of the following conditions: unrelieved persistent renal colic, febrile urinary tract infections, sepsis, or obstructive uropathy. Urinary drainage was achieved via the placement of a ureteral stent or by percutaneous nephrostomy (PNL).

### Statistical analysis

Continuous variables are presented as medians with inter-quartile range (IQR) and were compared using the Mann–Whitney U test. Categorical variables were compared using the Chi-squared test. Receiver operator characteristic (ROC) curve analysis was carried out with MedCalc® statistical software(version 15.2.2). Logistic regression was used to perform multivariable analysis when predicting the need for surgical intervention. The nomogram was created using R software(version 3.6.3). A two-tailed *p* value < 0.05 was considered to be statistically significant. All statistical analyses were performed using Statistical Package for the Social Sciences (SPSS) version 26 for Windows.

## Results

A total of 242 consecutive pregnant patients who attended our Emergency Department met the inclusion criteria for renal colic. After excluding patients with unknown interventional status (n = 9), missing data (n = 13), back pain or abdominal pain that was not caused by renal colic (n = 10), we recruited a final cohort of 212 patients. Of these, 100 patients (47.2%%) underwent surgical intervention while 112 patients (52.8%) were treated effectively by conservative management.

The demographic, clinical, laboratory, and imaging variables for the entire cohort, and separated by group, are given in Table 1. There were no statistically significant differences between the groups with regards to age (29 years *vs* 29 years, *p* = 0.71), gestation (22 weeks *vs* 24 weeks, *p* = 0.48), or BMI (21.9 kg/cm^2^ *vs* 22.55 kg/cm^2^, *p* = 1.00).

**Table 1:** Univariate analysis of demographic, clinical, laboratory, and ultrasound variables

Variables	Total (n = 212)	Surgery (n = 100)	No surgery (n = 112)	*p*
Age (Q1, Q3), years	29 (26, 32)	29 (26, 32)	29 (27, 32)	0.71
BMI (Q1, Q3), kg/cm^2^	22 (20.13, 25)	21.9 (20.6,25)	22.55 (19.85, 25.52)	1.00
Gestation (Q1,Q3), wk	23 (18, 27)	22 (19,27)	24 (17, 28)	0.48
History of stones, n (%)				
Yes	48 (22.6%)	26 (26%)	22 (19.6%)	0.33
No	164 (77.4%)	90 (74%)	90 (80.4%)	
Pain duration (Q1, Q3), d				< 0.01
< 4d	160 (75.5%)	60 (56%)	100 (89.3%)	
≥ 4d	52 (24.5%)	40 (44%)	12 (10.7%)	
Fever, n (%)				0.01
Yes	34 (16%)	23 (23%)	11 (9.8%)	
No	178 (84%)	77 (77%)	94 (90.2%)	
WBC s (Q1, Q3), 10^9^ /L	11.9 (10.05, 14.62)	12.6 (10.4, 14.92)	11.27 (9.53, 13.72)	0.01
CRP	1.15 (0.56, 3.12)	2.03(0.85, 3.83)	0.84 (0.40, 1.52)	< 0.01
Kidney stone size				0.83
< 1 cm	188(88.6%)	88 (88%)	100 (89.3%)	
≥ 1 cm	24 (11.4%)	12 (12%)	12 (10.8%)	
Ureteral stone size				< 0.01
< 8 mm	154 (72.6%)	54 (54%)	100 (89.3%)	
≥ 8 mm	58 (27.4%)	46 (44%)	12 (10.7%)	
Hydronephrosis (Q1, Q3), mm	16 (12, 30.75)	26.5 (15, 59.25)	13 (9.25, 19)	< 0.01
None/mild	158 (74.5%)	54 (54%)	104 (92.9%)	
Moderate/severe	54 (25.5%)	46 (46%)	8 (7.1%)	
Stone location, n(%)				< 0.01
Kidney	54 (25.5%)	20 (20%)	34 (30.4%)	
Ureter	92 (43.4%)	56 (56%)	36(32.1%)	
Proximal	52 (24.5%)	36 (36%)	16 (14.3%)	
Distal	40 (18.9%)	20 (20%)	20 (17.9%)	
None	66 (36.1%)	24 (24%)	42 (37.5%)	

*IQR* interquartile range, *BMI* body mass index, *wk* week, *d* day, *WBCs* white blood cells, *CRP* C-reactive protein

Data arising from the univariate analysis of clinical, laboratory, and imaging parameters, is provided in Table 1. There were significant differences between the two groups with respect to the duration of pain, fever, WBC count, CRP level, ureter stone size, hydronephrosis, and stone location.

**Fig. 1:**
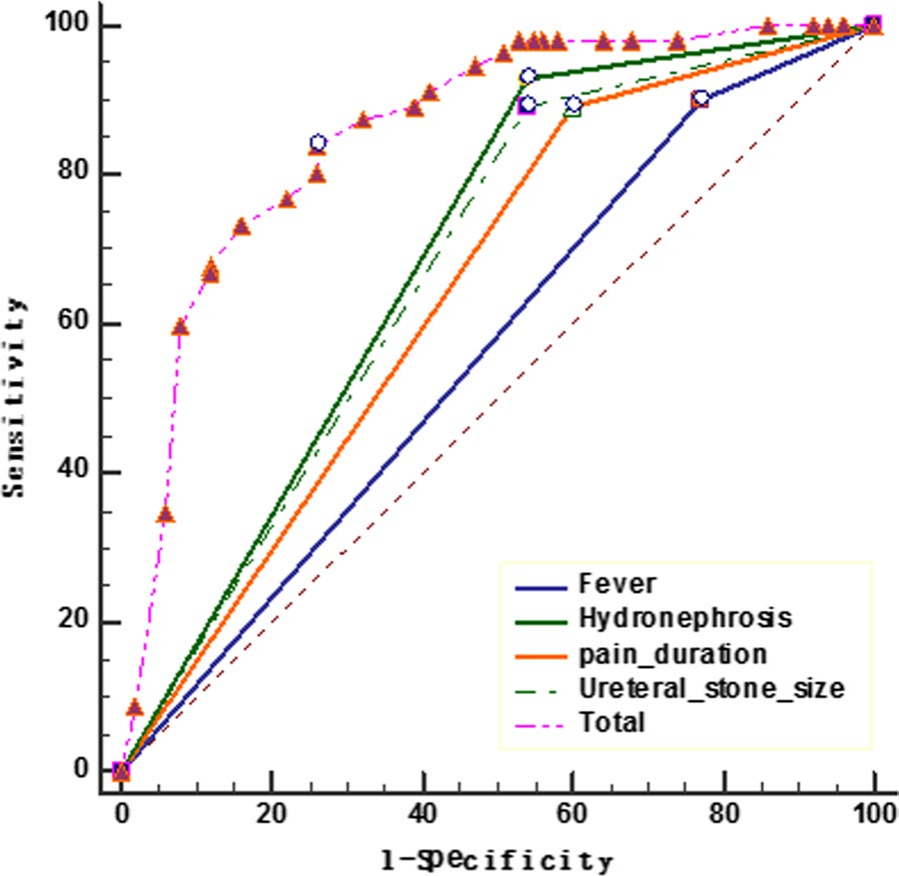
ROC curve analysis of risk factors (area under the curve [AUC] = 0.859, *p* < 0.01)

Table 2 shows the association between seven different variables and surgical intervention, as determined by univariate analyses. The use of these clinical variables in logistic regression analysis further yielded a model of 4 variables that showed good overall accuracy for identifying cases that require surgical intervention. The odds ratios (ORs) for each variable, adjusted for the other variables in the model, are shown with 95% confidence intervals (CIs). The area under the ROC curve was 0.859 (95% CI 0.81–0.90; *p* < 0.001) (Fig. 1).

**Table 2:** Multivariate analysis of significant variables identified by univariate analysis

Variable	Category	OR	95% CI	*p*
Hydronephrosis	None/mild *vs.* Moderate/Severe	9.9	3.9–25.2	< 0.01
Pain duration (d)	< 4 vs. ≥ 4	7.4	3.1–17.8	< 0.01
Ureteral stone size (mm)	< 8 vs. ≥ 8	8.1	2.6–25.4	< 0.01
Fever	Yes *vs.* No	2.8	1.0–7.7	0.04

OR, odds ratio; CI, confidence interval

**Fig. 2:**
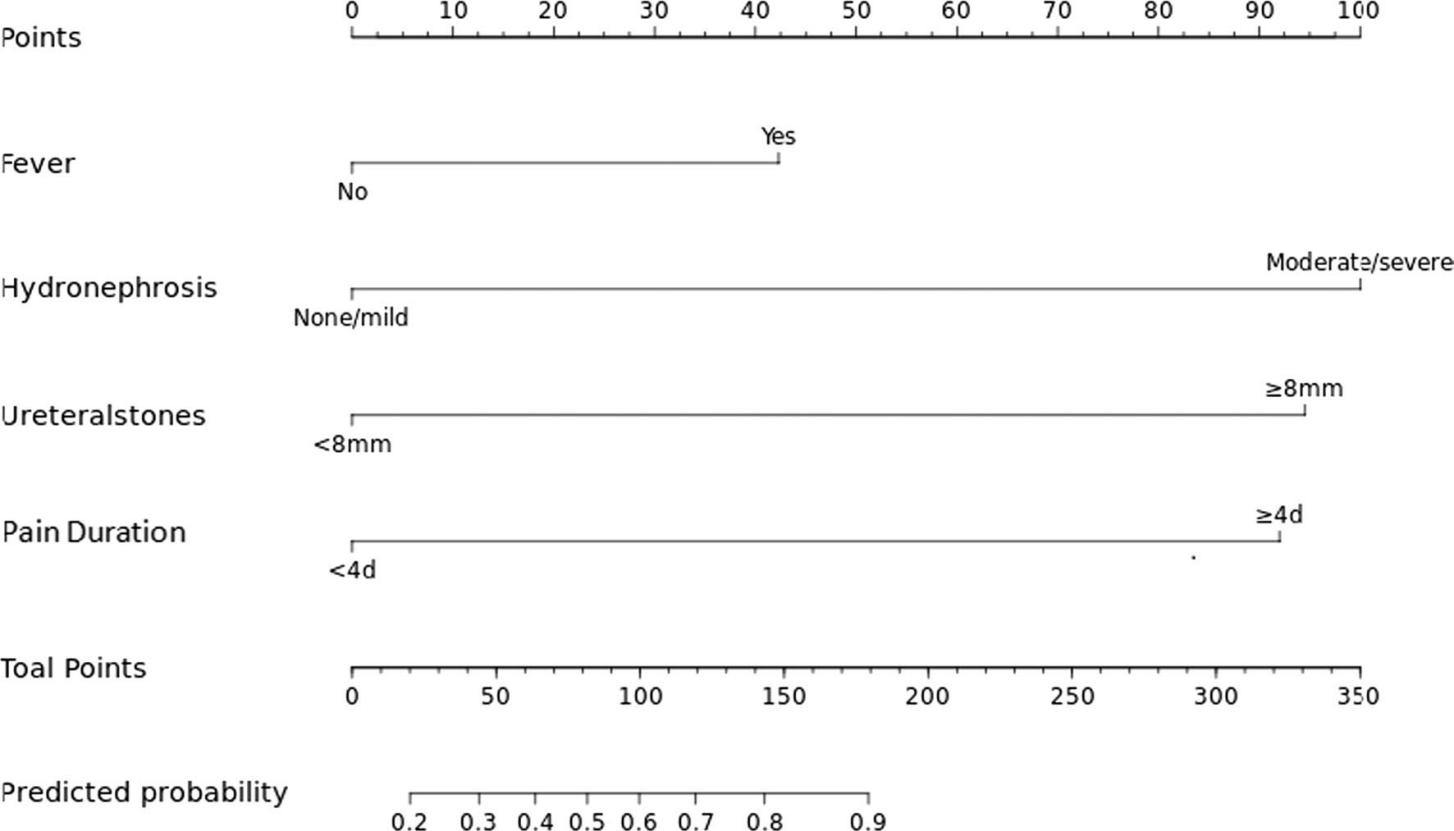
Nomogram for predicting surgical intervention in pregnant women suffering from renal colic. Points: score for each variable; Fever: Body temperature over 37.5 °C; hydronephrosis (mm): was classified as none/mild and moderate/severe; Ureterstones: Ureteral stones size larger or smaller than 8 mm by ultrasound; Pain Duration (d): time from the beginning of pain to attendance at the emergency room; total points: the total score of adding each variable score; Predicted probability: the likelihood of requiring surgical intervention

Next, we incorporated significant variables from the logistic regression analysis into R software in order to create a nomogram (Fig. 2).

The nomogram can be used to calculate a given patient’s risk score, thus identifying whether the patient is likely to require surgical intervention. For example, a pregnant woman with renal colic has no fever, and the time from pain to admission is 4 h. The B-ultrasound indicates that the ureteral stone size is 10 mm and the hydronephrosis is 44 mm. The corresponding score of this patient is 0 + 100 + 90 + 0 = 190, the predicted probability of requiring surgical intervention is 90%. If any one of these variables is present, then the sensitivity and specificity of the model is 74% and 83.93%, respectively.

## Discussion

We analyzed data from patients with renal colic during pregnancy who were treated in our department over the past 10 years. The main feature of this study is that we specifically evaluated patients with renal colic during pregnancy with a potential need for surgical intervention. We demonstrated that the duration of pain, ureteral stone size, hydronephrosis, and fever were independent predictors for surgical intervention.

The consideration of patient factors is an important element when making treatment decisions for renal colic [13]. In accordance with previous reports [14–16], our present analysis demonstrated that the duration of pain symptoms is a significant clinical predictor for surgical intervention. We found that patients with < 4 days of pain were more likely to be treated effectively by conservative techniques, while those with ≥ 4 days of pain were more likely to undergo surgical intervention. In fact, it has been suggested that delayed presentation, when considered as a single factor, may reduce the likelihood of stones passing spontaneously [17]. We consider that the duration of pain is an important predictor for surgical intervention as this is a relatively easy parameter to acquire in an era were significant emphasis is placed on imaging and laboratory findings. In addition, the intensity of pain is closely related to surgical intervention [18]. Unfortunately, we were unable to acquire comprehensive evaluation records that related to the intensity of pain.

The occurrence of hydronephrosis in a normal pregnancy has been attributed to hormonal effects, external compression, and intrinsic changes in the ureteral wall [19]. Pathological hydronephrosis can usually be distinguished from physiological hydronephrosis by the presence of flank pain or the radiographic or sonographic visualization of the cause of the obstruction. In the present study, the degree of hydronephrosis differed between the two groups and therefore influenced the decision as to whether to perform surgery. Furthermore, we found that patients with moderate to severe hydronephrosis are more likely to undergo surgical intervention in pregnancy.

Ureteral stone size is a well-recognized prognostic factor for the successful passage of stones and is a key factor to consider when decision making [10, 19]. The passage rates for stones < 3 mm, 3–4.9 mm, 5–6.9 mm, and ≥ 7 mm have been reported to be 50%, 13%, 10%, and 0%, respectively [20]. In the present study, we found that a stone size stones ≥ 8 mm is a risk factor for surgical intervention. Compared with non-pregnant women, ureteral stones are easier to pass during pregnancy due to the fact that the ureters are dilated.

Approximately 50% of pregnant women with stones will suffer from urinary tract infection and will require antibiotics. Urgent decompression of the urinary system in a febrile or septic pregnant woman, followed by a subsequent definitive solution under improved conditions, is highly recommended for such cases [21]. In the present study, 16% of pregnant women had fever and 70% patients who had a persistently high fever underwent surgery. However, antibiotic treatment was effective for pregnant women who had not undergone surgery and had transient fever. Through ROC curve, fever can predict that 56.6% of patients will require surgery. Therefore, a persistent high fever in pregnant women with acute renal colic may result in sepsis and systemic inflammatory response syndrome, thus endangering the safety of the mother and child; surgical intervention is vital in such cases.

Our analyses ultimately allowed us to create a nomogram. Based on this nomogram, it is possible to calculate a risk score for a given patient. Thus, we can determine the probability of surgical intervention. We believe that the use of this nomogram in clinical practice may help us to guide the management of patients suffering from renal colic in pregnancy.

There are several limitations to our study that should be considered. Owing to the retrospective nature of the data acquisition, the decision to intervene surgically lacks appropriate standardization. Moreover, the decision to treat patients non-surgically was not standardized and we cannot therefore assess all of the potential factors that were involved in the decision-making process. Another limitation is that this analysis reflects our own treatment decisions that may not be in concordance with other urological centers. We are based in the South China Urology Stone Treatment Center. Many of our patients had undergone a series of interventions in an outer hospital before coming to our institution. This may have increased our rate of surgical intervention. However, none of our patients were found to have elevated levels of serum creatinine. Furthermore, except for one case, involving premature delivery after the placement of a ureteral stent, all patients were discharged from the hospital safely. Although one patient with premature birth occurred after surgery, the patient also had other important factors leading to premature birth, such as fever, pyelonephritis, which could led to adverse results*.* At our institution, ultrasound is the imaging study of choice. This is because ultrasound can safety evaluate stone size, stone location, and hydronephrosis in pregnancy. Although computed tomography (CT) has become the preferred imaging method for diagnosing urinary stones, we only use B-ultrasound in pregnant cases. Data from CT imaging techniques are likely to improve the estimation of calculus size and location, particularly when very small calculi are present.

## Conclusion

Using local data, we identified several independent predictors for surgical intervention during an episode of renal colic in pregnancy; we used this information to create a nomogram. Our analyses identified that a duration of pain ≥ 4 days, a stone size ≥ 8 mm, fever, and hydronephrosis, all play significant roles in the prediction of surgical intervention. Our nomogram may have clinical utility when making decisions regarding treatment options. An electronic version of this nomogram will be available in the future.

## Data Availability

The datasets used and/or analysed during the current study are available from the corresponding author on reasonable request.

## Abbreviations

ED: Emergency Department
ROC: Receiver operating characteristic
BMI: Body mass index
WBCs: White blood cells
CRP: C-reactive protein
